# Clinical effects of mindfulness meditation and cognitive behavioral therapy standardized for insomnia: A protocol for a systematic review and meta-analysis

**DOI:** 10.1097/MD.0000000000013499

**Published:** 2018-12-21

**Authors:** Moon Joo Cheong, Go-Eun Lee, Hyung Won Kang, Sooim Kim, Hye Kyung Kim, Han-ik Jo, Yeonseok Kang, Jae-Hyo Kim, Hyeon-Gi Baek

**Affiliations:** a Education Graduate of Hanyang University; b Department of Oriental Rehabilitation Medicine, Korean National Rehabilitation, Seoul; c Department of Korean Neuropsychiatry Medicine & Inam Neuroscience Research Center, Wonkwang University Sanbon Hospital, Gunpo; d Department of Clinical Counseling Psychology, CHA University; e Department of Counseling Psychology, Hankuk University of Foreign Studies, Seoul; f Department of Medical History; g Department of Meridian & Acupoint, College of Korean Medicine; h The Institute of Mind Humanities, Wonkwang University, Iksan, Republic of Korea.

**Keywords:** cognitive behavioral therapy, insomnia disorder, mindfulness meditation, protocol, systematic review

## Abstract

**Introduction:**

This systematic review protocol describes the methods that will be used to evaluate the efficacy and safety of mindfulness meditation and cognitive behavioral therapy programs as a psychological intervention for insomnia disorders.

**Methods and analysis:**

We will search the following 11 electronic databases without language or publication status restrictions: MEDLINE, the Cochrane Central Register of Controlled Trials, EMBASE, Allied and Complementary Medicine Database, Cumulative Index to Nursing and Allied Health Literature, and PsycARTICLES. Furthermore, we will also search 5 Korean-language databases (Oriental Medicine Advanced Searching Integrated System, Korean studies Information Service System, Research Information Service System, Korean Medical Database, and Korea Citation Index). The study selection and data extraction will be performed independently by 2 authors. The study quality assessment and evaluation of the quality of evidence for the main findings will be performed independently by 2 authors using the Cochrane tool for assessing risk of bias and predefined criteria (the Grading of Recommendations Assessment, Development, and Evaluation approach). Data synthesis and analysis will be performed using RevMan Version 5.3. Data will be synthesized by either a fixed effects or random effects model according to a heterogeneity test or the number of studies included in the meta-analysis. If any plan for documenting important protocol amendments changes, the researchers will have a revision agreement and then register the modification in the International Prospective Register of Systematic Reviews (PROSPERO).

**Ethics and dissemination:**

Ethical approval will not be required because individual patient data are not included and because this protocol is for a systematic review. The findings of this systematic review will be disseminated through conference presentations.

PROSPERO registration number: CRD42018111217.

## 1 Introduction

Healthy sleep for humans restores function and vitality, promotes memory integration, and maintains immune function. Nonetheless, 10% to 12% of the world's population experience sleep disorders because of a variety of causes.^[1]^ In Korea, the number of patients with insomnia has increased by 40.19% over the past 5 years^[2]^ and the average sleep time in Korea was 7 hours and 41 minutes, the lowest among Organisation for Economic Co-operation and Development countries.^[3]^ Individuals who experience sleeplessness more than 3 times a week now account for over 17.0% of the population.^[1,4]^

According to National Health Insurance Corporation data, the number of patients who have been treated for insomnia for the past 5 years (2013–2017) has exceeded 2 million, and the total cost of the treatment was estimated to be about 204.6 billion won in Korea.^[4]^ This is not very different from other countries.^[5,6]^ According to the National Sleep Foundation Survey, from 2013 to 2015, 51% of US adults have at least one symptom associated with insomnia.^[7]^ The US also has an annual expenditure of $107 to $920 billion for treating insomnia.^[8,9]^ In Canada, it was found to cost $5000 per treatment.^[10]^ As such, insomnia has a high burden for the individual and society because of the high cost of evaluation and treatment. Nonetheless, patients who currently have insomnia are predominantly prescribed medications that can be abused by patients with chronic insomnia or can increase resistance-dependence, creating problems other than insomnia.^[11]^ Untreated insomnia also increases the risk of heart disease, motor vehicle accidents, memory disorders, depression, and dysfunction.^[12,13]^ Without appropriate intervention, it can last for years after its development and the probability of other comorbid psychiatric disorders increases.^[14,15]^

Recently, a variety of programs using a psychological intervention have been use to increase the quality of life of patients with insomnia and to reduce the adverse effects of drugs in place of simple drug prescriptions.^[16]^ Some patients experiencing sleeplessness may also have psychological problems with a behavioral aspect such as insomnia.^[17]^ Typical psychological intervention programs include cognitive behavioral therapy (CBT)^[18,19]^ and meditation programs^[20,21]^ based on mindfulness, which are widely used and have demonstrated their efficacy in many places.

In particular, CBT aims to reduce the cognitive physiologically elevated arousal level, correct maladaptive sleep habits, and modify dysfunctional beliefs and attitudes toward sleep.^[18]^

Thus, recently, many patients with insomnia have a preference for CBT over drug therapy with its risk of increasing dependence and side effects. In addition, the mindfulness-based meditation (MM) program,^[20,21]^ Kabat-Zinn's “Mindfulness-Based Stress Reduction” (MBSR),^[22,23]^ and meditation on sleep disturbances in cancer patients has been shown to improve sleep quality^[24]^ and efficiency. Through a comparative study of medication- and mindfulness meditation use in groups of cancer patients,^[25]^ it has been reported that meditation has a more positive effect on the improving sleep quality relative to drug treatment. In addition, research on mindfulness, well-being, and sleeping has consistently proven that mindfulness has a significant influence on improving sleep quality and quality of life.^[26]^

Therefore, this study will systematically examine the effects of psychological treatment interventions, such as CBT and MM therapy, which both demonstrate that the mind is correlated with behavior, on sleep disorders. A systematic review of psychiatric interventions on existing insomnia disorders was conducted primarily on insomnia disorders and the effects of CBT programs before the Diagnostic and Statistical Manual of Mental Disorders, Fifth Edition (DSM-5) was published in 2014.^[14,27,28]^ In addition, subjects who undergo MM therapy are broadly classified as having mental disorders,^[29]^ and there is limited research that clearly defines the subject as having a sleep disorder or insomnia. Thus, there is a lack of a systematic review of the effectiveness of mindfulness meditation (MM) and CBT, which is a psychological intervention program that modifies behavior by controlling the mind. This study will investigate the clinical efficacy of programs of standardized MM and CBT for solving the sleep disorders using findings studies published by June 2018.

We believe that the results of this systematic review and meta-analysis will help clinicians optimize treatment protocols for patients with insomnia introduce safe and effective insomnia disorders treatment strategies for use in clinical settings.

## 2 Methods and analysis

### 2.1 Study registration

The protocol for this systematic review has been registered in the International Prospective Register of Systematic Reviews (PROSPERO) (registration number, CRD42018111217) on October 10, 2018. We will conduct a systematic review according to this protocol, but if protocol amendments occur, the dates, changes, and rationale for each amendment will be tracked in PROSPERO. This protocol is reported in accordance with the Preferred Reporting Items for Systematic Review and Meta-Analysis Protocols 2015 statement^[30]^ and the Cochrane Handbook for Systematic Reviews of Interventions.^[31,32]^

### 2.2 Data sources and search strategy

The research strategy using domestic and foreign databases will be conducted by 2 researchers and methodologists. Six English-language databases (MEDLINE via PubMed, EMBASE via Elsevier, the Cochrane Central Register of Controlled Trials, the Allied and Complementary Medicine Database via EBSCO, the Cumulative Index to Nursing and Allied Health Literature via EBSCO, and PsycARTICLES via ProQuest) and 5 Korean-language databases (Oriental Medicine Advanced Searching Integrated System, Korean studies Information Service System, Research Information Service System, Korean Medical Database, and Korea Citation Index will be searched.

We will also search the reference lists of the relevant articles and perform a manual search on Google Scholar to identify any potential additional studies. We will also include Standard and Ideal as well as Core within the search range suggested by the COSI model proposed by the National Library of Medicine. According to the COSI model, we will include not only the literature published in journals but also “gray literature” such as theses and conference proceedings. There will be no language restriction and the period will be set from 2008 to August 2018.

The search terms will be composed of the participant term part (e.g., “Insomnia disorder and Insomnia”) and the intervention term part (e.g., Mindfulness or MM or Cognitive Behavior Therapy or CBT or MBSR^[33,34]^ or MCBT). The search strategies for MEDLINE and EMBASE are shown in Table 1 and will be modified and used similarly for the other databases.

**Table 1:**
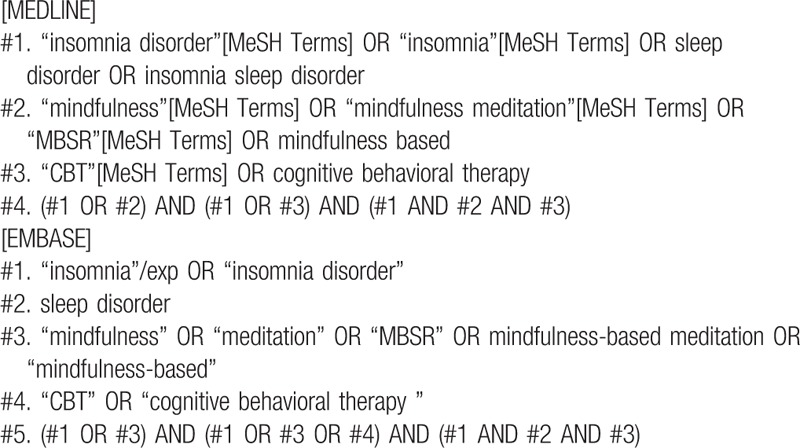
Search strategies for the MEDLINE and EMBASE.

### 2.3 Inclusion criteria

#### 2.3.1 Types of studies

We will only include randomized controlled trials (RCTs). Studies using inappropriate random sequence generation methods such as alternate allocation or birth day will be excluded. If only the expression “randomization” (that the heart governs the mind) is mentioned without the randomization methods, it will be considered an RCT and will be included in this review.

This study will include systematic review studies, in which structured MM programs and CBT programs are conducted for at least 6 weeks and medical outcomes such as sleep quality and sleep time are present. However other designs such as in vivo, in vitro, case reports or studies, retrospective studies, qualitative studies, and uncontrolled trials will be excluded, as will all studies that fail to provide detail results.

In brief, the search strategies using the patient/participants/population/problem, intervention, comparison, outcome (PICO) and timing (setting, setting, and study design) approach are shown in Table 2.

**Table 2:**
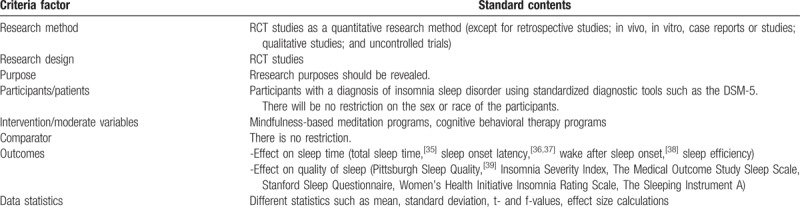
The studies analytical criteria factors and framework (search strategies using PICO).

#### 2.3.2 Types of participants

We will include studies on patients, aged 20 to 60 years, with a diagnosis of insomnia or sleep disorders ^[40]^ using standardized diagnostic tools such as the DSM-5. There will be no restriction on the sex or race of the participants. However, studies will be excluded if the participants have other serious illnesses, such as severe physical illness or findings suggesting a medical condition (e.g., abnormal clinical laboratory test, ECG, Chest PA, and vital sign) such as Parkinson's disease.

#### 2.3.3 Types of interventions

The intervention methods included in this study are standardized MM programs and CBT programs. There are no other restrictions regarding the control intervention.

#### 2.3.4 Types of outcome measures

The primary outcome measures are as follows:

Primary care outcomes were reported using the following measures of change in sleep time and sleep quality:

a. Effect on sleep time
  1. An activity recorder (Actigraphy)^[41]^ that records activity during sleep worn on the wrist or ankle
  2. Polysomnography (^[42]^ test, which is a medical examination that measures the physiological changes by measuring EEG ^[43]^, EMG, EEG, ECG, snoring, and respiration, that is used to diagnose a disorder
  3. A sleep diary is used to measure the patient's sleep habits, sleep hygiene, and sleep problems for 2 weeks and to evaluate the progress of the treatment.
  We will measure the following sleep variables: total sleep time,^[44,45]^ sleep onset latency,^[36,37,45]^ wake after sleep onset, and sleep efficiency^[46]^ by using the above tools.
b. Effect on quality of sleep
  (1) Pittsburgh Sleep Quality^[39,47]^ is a questionnaire for measuring sleep quality and discomfort for the past month. It consists of 7 subfactors and scores from 0 to 21 points.
  (2) Insomnia Severity Index was developed to assess insomnia and is a 5-point cut-off self-report scale consisting of a total of 7 items. A higher score represents more severe insomnia.
  (3) The Medical Outcome Study Sleep Scale is composed of 12 items, measured in the range of 0 to 100, and the lower the score, the better the quality of sleep.
  (4) Stanford Sleep Questionnaire is a 7-level scale with subjective sleepiness ranging from 1 to 7, with lower scores indicating improved sleep quality.
  (5) Women's Health Initiative Insomnia Rating Scale consists of a 5-item scale for evaluating subjective sleep quality for sleep initiation and maintenance, the lower the score, the better the quality of sleep.
  (6) The sleeping instrument A consists of 15 items and measures 4 points. Ranging from a minimum score of 15 to a maximum score of 60, with higher scores representing better sleep.

The secondary outcome measures are as follows:

Secondary medical outcomes including depression, stress, anxiety, decreased fatigue, and quality of life measured using the Hamilton Depression Scale,^[48]^ Beck Depression Inventory,^[49]^ Perceived Stress Scale,^[50,51]^ State-Trait Anxiety Inventory State Version,^[52]^ Quality of life as measured by validated assessment tools such as the Stroke Specific Quality of Life Scale,^[53]^ 36-Item Short Form Health Survey,^[54]^ and Fatique-FSI.^[55]^

### 2.4 Study selection

The study selection will be conducted by 2 independent researchers according to the aforementioned selection criteria. After removing duplicates, we will review the titles and abstracts of the searched studies for relevance and then evaluate the full texts of the remaining studies for eligibility. Any disagreement on study selection will be resolved through discussion with other researchers and reviewers. The literature selection process will be reported in accordance with the PRISMA guidelines.^[56]^

### 2.5 Data extraction

Two independent researchers, using a standardized data collection form, will perform and cross check the data extraction. Discrepancies will be resolved through discussion with other researchers. The extracted items will include the first author's name; year of publication; country; sample size and number of dropouts; details about the participants, intervention, and comparisons or control; duration of the intervention and main outcomes; and adverse effects. Two independent researchers will then organize and code the extracted data using Excel 2007 (Microsoft, Redmond, WA) that will then be shared among researchers using Dropbox (Dropbox, Inc., CA) folders. If the obtained data are insufficient or ambiguous, we will contact the corresponding authors of the included studies via e-mail to request additional information.

### 2.6 Quality assessment

#### 2.6.1 Assessment of risk of bias

We will independently assess the risk of bias using Cochrane's assessment of risk of bias.^[57]^ Two independent researchers will assess the methodological quality of the included studies and the quality of evidence for each main finding. Discrepancies will be resolved through discussion with other researchers.

To assess the evidence of RCT-based studies, we will assess the random sequence generation, allocation concealment, blinding of participants and personnel, blinding of outcome assessments, incomplete outcome data, selective reporting, and other biases for each included study. Each domain will be categorized into one of 3 groups: “yes means low risk,” “unclear means insufficient information,” and “no means high risk.” Each evaluation will be recorded in an Excel 2007 (Microsoft) spreadsheet and will be shared among researchers in Dropbox (Dropbox Inc) folders. The evaluated results will be presented in a full review using Review Manager Version 5.3 (Cochrane, London, UK). The list used for the assessment of the risk of bias is shown in Table 3.

**Table 3:**
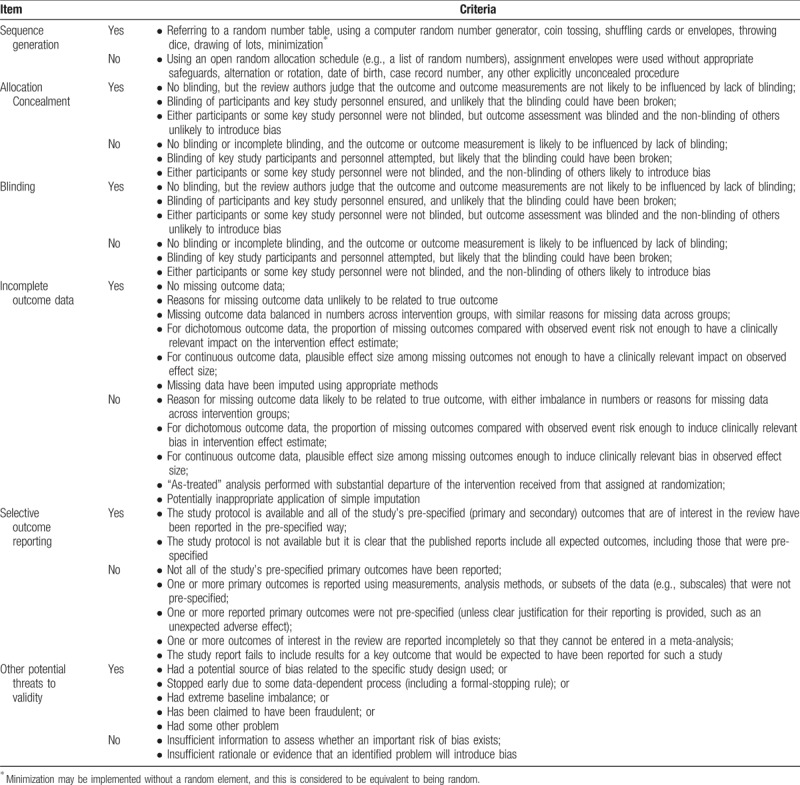
Cochrane's assessment of risk of bias.

### 2.7 Data synthesis and analysis

The data synthesis and analysis will be performed using Review Manager Version 5.3 and CMA 2.0 (Comprehensive Meta-Analysis software 2.0) and shared among researchers in Dropbox folders. Descriptive analyses of the details of the participants, interventions, and outcomes will be conducted for all included studies. A quantitative synthesis will be performed if there are studies using the same types of intervention, comparison, and outcome measures. The data will be pooled as the mean difference or standardized mean difference with 95% confidence intervals (CI s) for continuous outcomes. The heterogeneity of effect sizes will be assessed using the I-squared statistic. We will consider I-squared values greater than 50% and 75% indicative of substantial and high heterogeneity, respectively. In the meta-analyses, a random effects model will be used when the heterogeneity is significant (an I-squared value >75%), while a fixed effects model will be used when the heterogeneity is non-significant. A fixed effects model will be also used when the number of studies included in the meta-analysis is very small, where inter-study variance estimates have poor accuracy.^[58,59]^ When it is believed that the heterogeneity is too high for the results to be synthesized (an I-squared value >75%), a subgroup analysis will be conducted as follows to determine the cause of heterogeneity.

### 2.8 Subgroup analysis

If the heterogeneity is evaluated as significant (an I-squared value >75%) and the necessary data are available, we will conduct a subgroup analysis to account for the heterogeneity. A subgroup analysis will be conducted according to the following criteria:

(1) the intervention program kind;
(2) the intervention program period and time; and
(3) controls and comparators.

### 2.9 Sensitivity analysis

To identify the robustness of the meta-analysis result, we will perform sensitivity analyses by determining the effects of excluding

(1) studies with high risks of bias,
(2) studies with missing data, and
(3) outliers.

### 2.10 Assessment of reporting bias

If there are more than 10 trials included in the analysis, reporting biases such as publication bias will be assessed by using funnel plots. When reporting bias is implied by funnel plot asymmetry, we will attempt to explain possible reasons.

## 3 Ethics and dissemination

Ethical approval will not be needed because the data used in this systematic review will not be individual patient data and there will be no concerns regarding privacy. The results will be disseminated by the publication of a manuscript in a peer-reviewed journal or presentation at a relevant conference.

## 4 Discussion

Insomnia disorder is one of the most common illnesses in modern populations. If insomnia persists, it causes a decline in productivity and quality of life.^[60]^ Thus, if patients with insomnia are not adequately treated,^[15,61]^ it may persist for years after onset.^[62]^ The patients who are treated for insomnia in clinical practice are mainly prescribed medication.^[61]^ However, the drug prescribed should be considered with the patient's characteristics, such as the cause, duration, and intensity of the patient's insomnia. Long-term treatment with medication may also increase patient dependence.^[63]^ Therefore, the sleep disorder is an involuntary disease that cannot be cured by medication but cannot be controlled by the will of the individual and it is changing the perception that it is a disorder that requires therapeutic intervention. And in clinical view, it has avoided the side effects of drugs and increased the use of various programs using psychological interventions in a safe environment.

Therefore, recently, the number of studies on MM and CBT, psychological interventions used to treat insomnia disorder has increased.^[14,19]^ In addition, this psychological intervention can actually prove and show the correlation between psychology and behavior, that is, human cognition, behavior, and emotion.^[64]^ The purpose of this study is to investigate the clinical efficacy of standardized MM and CBT^[47,65,66]^ programs for solving the sleep problems of the subjects with insomnia using data from studies published by June 2018. We believe that the results of this systematic review will enable clinicians to optimize treatment protocols for patients with insomnia and to introduce safe and effective sleep disorder treatment strategies for use in clinical settings. The results of this review will also provide a broader evidence base for psychological intervention beside pharmacotherapy for patients with insomnia. In addition, policy makers will be able to confirm the effectiveness of MM and CBT^[47]^ programs as therapeutic interventions, which are currently covered by public insurance. Finally, it is expected that it will be possible to provide basic data for the scientific theory of ShimJuShinJi (that the heart governs the mind),^[64]^ demonstrating the correlation between mind and body, which is the viewpoint of Oriental medicine.

## Author contributions

The study was conceptualized by CMJ. The protocol was drafted by CMJ and KHW. The search strategy was developed by CMJ, KHW, and LGE. LGE, KSI, KHK, JHI, KYS, and KJH revised the manuscript. BHG submitted the manuscript for publication. All authors have read and approved the final manuscript.

**Conceptualization:** Moon Joo Cheong, Go-Eun Lee, Hyung Won Kang.

**Data curation:** Moon Joo Cheong.

**Formal analysis:** Moon Joo Cheong.

**Funding acquisition:** Hyung Won Kang.

**Investigation:** Moon Joo Cheong.

**Methodology:** Moon Joo Cheong.

**Resources:** Moon Joo Cheong, Hyung Won Kang.

**Software:** Moon Joo Cheong.

**Validation:** Moon Joo Cheong.

**Visualization:** Moon Joo Cheong.

**Writing – original draft:** Moon Joo Cheong, Hyung Won Kang.

**Writing – review & editing:** Moon Joo Cheong, Go-Eun Lee, Hyung Won Kang, Sooim Kim, Hye Kyung Kim, Han-ik Jo, Yeonseok Kang, Jae-Hyo Kim, Hyeon-Gi Baek.

Hyung Won Kang orcid: 0000-0001-6497-0100.
